# Connectomics-Based Functional Network Alterations in both Depressed Patients with Suicidal Behavior and Healthy Relatives of Suicide Victims

**DOI:** 10.1038/s41598-019-50881-y

**Published:** 2019-10-04

**Authors:** Gerd Wagner, Feliberto de la Cruz, Stefanie Köhler, Fabricio Pereira, Stéphane Richard-Devantoy, Gustavo Turecki, Karl-Jürgen Bär, Fabrice Jollant

**Affiliations:** 1 Department of Psychiatry and Psychotherapy, Jena University Hospital, Philosophenweg 3, 07743 Jena, Germany; 2 McGill group for Suicide Studies, McGill University & Douglas Mental Health University Institute, Montréal, Canada; 3 Université Paris-Descartes, Faculté de médicine & Clinique des Maladies Mentales et de l’Encéphale (CMME), Hôpital Sainte-Anne, Paris, France; 4 Departments of Radiology & Psychiatry, University Hospital Center of Nîmes, Nîmes, France

**Keywords:** Functional magnetic resonance imaging, Neural circuits, Depression

## Abstract

Understanding the neural mechanisms of suicidal behavior is crucial. While regional brain alterations have previously been reported, knowledge about brain functional connectomics is currently limited. Here, we investigated differences in global topologic network properties and local network-based functional organization in both suicide attempters and suicide relatives. Two independent samples of depressed suicide attempters (N = 42), depressed patient controls (N = 43), healthy controls (N = 66) as well as one sample of healthy relatives of suicide victims (N = 16) and relatives of depressed patients (N = 16) were investigated with functional magnetic resonance imaging in the resting-state condition. Graph theory analyses were performed. Assortativity, clustering coefficients, global efficiency, and rich-club coefficients were calculated. A network-based statistic approach was finally used to examine functional connectivity matrices. In comparison to healthy controls, both patient groups showed significant reduction in assortativity, and decreased functional connectivity in largely central and posterior brain networks. Suicide attempters only differed from patient controls in terms of higher rich-club coefficients for the highest degree nodes. Compared to patient relatives and healthy controls, suicide relatives showed reduced assortativity, reduced clustering coefficients, increased global efficiency, and increased rich-club coefficients for the highest degree nodes. Suicide relatives also showed reduced functional connectivity in one anterior and one posterior sub-network in comparison to healthy controls, and in a largely anterior brain network in comparison to patient relatives. In conclusion, these results suggest that the vulnerability to suicidal behavior may be associated with heritable deficits in global brain functioning – characterized by weak resilience and poor segregation - and in functional organization with reduced connectivities affecting the ventral and dorsal prefrontal cortex, the anterior cingulate, thalamus, striatum, and possibly the insula, fusiform gyrus and the cerebellum.

## Introduction

Suicide is a worldwide public health problem with approximately 800,000 victims per year and a leading cause of death in most societies^1^. In addition, 10 to 20 times more attempt suicide, and a history of such attempts is considered a major risk factor of future suicide death^2^. More than 90% of all suicide victims suffer from an adjacent psychiatric disorder, most commonly depression^3,4^. However, only a small minority of depressed persons will die from suicide^5^. Identifying depressed patients at risk of suicide is therefore crucial for developing sustainable and efficient preventive interventions. Unfortunately, the only clinical risk factor assessment available has poor predictive power^6^.

A growing number of relatively recent studies have investigated the underlying mechanisms of suicidal behavior using neuroimaging^7,8^. Both structural and functional neuroimaging studies have been conducted in individuals with *a history* of suicide attempts, shedding light on a potential role of the ventral and dorsal prefrontal regions, the anterior cingulate cortex, the temporal and parietal cortices, as well as selected subcortical nuclei, among others. Differences in brain activation or structure in suicide attempters vs. patient controls have notably been related to deficits in decision-making^9–11^ or social perception^11,12^. Moreover, recent studies have suggested that some deficits observed may be heritable, being found in close relatives of suicide victims who never attempted suicide^13^, which is in agreement with the known heritability of suicidal acts^14^. However, knowledge about alterations affecting the organization and functioning of brain networks in relation to suicidal behavior is much more limited.

In the field of functional neuroimaging, functional connectivity (FC) analyses of resting-state functional Magnetic Resonance Imaging (rs-fMRI) datasets is an established method for studying the FC of a specific brain region or the related network architecture^15–17^. Rs-fMRI allows examination of the tonic rather than phasic activation level underlying functional connectivity, which might be a more powerful way to identify intrinsic network abnormalities in a specific population. In contrast to task-based fMRI studies, rs-fMRI studies are not confounded by a subject’s motivation, present cognitive state or by specific task-related effects, such as the impact of practice or applied strategy, thus increasing the inter-subject and intra-subject reproducibility. Furthermore, as shown by Fox and Greicius^18^, resting state studies likely have a better signal to noise ratio than task-based fMRI studies. Thus, rs-fMRI is more apt to identify specific and reproducible markers of neural dysfunction associated with suicidal behavior.

An increasing number of pathological conditions have been associated with abnormal FC between particular brain regions or in network organizations^19^, providing potentially valuable information for understanding the pathophysiology of these disorders. Despite the relevance of FC analyses, so far, few rs-fMRI studies have been conducted in suicide attempters, and none in relatives. Two studies used the amplitude of low-frequency fluctuation (ALFF) method to explore abnormal resting-state brain activity^20,21^, and showed changes in ALFF values in the middle and superior temporal, ventromedial prefrontal, and occipital regions in suicide attempters compared to depressed controls. Using independent component analysis (ICA), Zhang *et al*.^22^ found increased FC in the cerebellum and the occipital cortex as well as decreased FC in the precuneus in adolescent depressed suicide attempters compared to depressed controls. Very recently, Kang *et al*.^23^ demonstrated abnormally increased FC between the amygdala (used as a seed region) and the insula, orbitofrontal cortex and middle temporal gyrus in adult suicide attempters with major depressive disorder (MDD) compared to MDD controls.

With the growing use of connectomics, we are now able to shift the view from a local connectivity level towards a global network perspective. Based on functional as well as structural data, altered network organizations in psychiatric disorders are thus revealed^24,25^. Advanced mathematical approaches such as graph theory provide comprehensive insights into the key organizational principles of brain networks (e.g. small-worldness) that support efficient neural processing. Graph theory has especially proven to be useful in the analysis of such data, providing multiple metrics to assess the topological properties of the underlying brain graphs^26^.

Recent studies have shown that healthy brain functioning is characterized by higher clustering and smaller shortest path lengths (i.e. higher global efficiencies) compared to a random network^27^, pointing toward both central features of a small-world configuration, i.e. segregated and integrated information processing. Previous connectome studies in patients with MDD and schizophrenia often exhibited abnormal small-world metrics for their respective brain networks^24,28^. *Assortativity*^29^ is a topological measure of network resilience and defined as a correlation coefficient between the degrees of all nodes on two opposite ends of a link. Moreover, the so-called *rich-club coefficient* describes the density of connectivity only between high-degree nodes (“hubs”) and has been assumed to indicate overall brain communication and resilience^30^. For example, abnormal rich-club organization has been reported in patients with schizophrenia^31^. Thus, these graph metrics provide information about the global organizational properties of functional and structural brain networks of a given group, as well as differences between groups^32^. A recently developed statistical method called the network-based statistic (NBS) yields additional information about differences in local connectivity, offering an effective way to deal with the multiple comparison problems arising in the analysis of seed-based connectivity^33^. To the best of our knowledge, there are currently no studies investigating alterations in global network functioning and local functional connectivity (using the NBS approach) in relation to suicidal behavior.

Thus, since suicidal behavior is characterized by a complex set of affective, cognitive and interpersonal dysfunctions^7^, the current study first aims at examining differences in the above-described global properties of all functional connections between brain regions, i.e. the connectome, using rs-fMRI data. We combined data from two independent samples of depressed patients with MDD, with and without a personal history of suicidal behavior, and healthy controls to increase the statistical power in order to investigate global connectome alterations. We further investigated a sample of first-degree biological relatives of suicide victims and relatives of depressed patients without a family history of suicide to uncover the heritable components of potential differences in the global network organization. We only included patients with MDD in the present study, because previous studies directly comparing patients with MDD, bipolar disorder and schizophrenia showed considerable heterogeneity with regard to structural abnormalities^34,35^ as well as with regard to alterations in functional connectivity^36^. The second aim of the study was to investigate differences in local FC to identify specific brain regions associated with suicidal behavior.

The two analytic approaches described above were employed: graph theory analyses were conducted to identify potential changes in global topologic properties. NBS was applied to test for connectome-wide differences in network connectivity (while controlling for multiple comparisons).

Due to the multifaceted nature of suicidal behavior, we hypothesized that the functional disorganization of resting-state brain networks would exist at both the global and local scales. We especially hypothesized that global parameters of the functional connectome could similarly differentiate MDD patients with and without a personal history of suicidal behavior, as well as healthy relatives of suicide victims from healthy relatives of patients with MDD without a family history of suicidal behavior. Based on previous studies in suicidal behavior, we expected to find differences in the brain networks implicated in impulsivity, social and emotional processing and decision-making, notably fronto-temporo-parieto-striatal structures.

## Methods and Materials

### Participants

#### Suicide attempters samples

As in our previous structural MRI study^37^, three groups of male and female participants aged 18–57 years were included in the present study from Montreal (Québec, Canada) and Jena (Germany) (Table 1): (1) currently depressed patients with a personal history of attempted suicide (*suicide attempters*); (2) currently depressed patients with no personal history of suicide attempt (*patient controls*); and (3) non depressed controls with no personal or first- or second-degree family history of suicidal behavior (*healthy controls*) (Table 1). All suicide attempters and patient controls were depressed at the time of scanning, as determined by the Hamilton Depression Rating Scale (HDRS-21) and fulfilled the criteria for a major depressive episode according to the Structured Clinical Interview for DSM-IV Axis I Disorders (SCID-I). In Montreal, outpatients were recruited from the Douglas Mental Health University Institute. Patients from Jena were recruited from the inpatient service of the department of Psychiatry and Psychotherapy at University Hospital Jena. Moreover, none of the participants were medicated at the time of scanning in Montreal, while all patients were on antidepressant medication in Jena. No significant differences in the kind of antidepressant medication in patients with vs. without suicidal behavior in the Jena sample were detected. In both samples, none of the healthy controls were taking any psychopharmacological medication.

**Table 1:** Demographic and Clinical Characteristics of the three samples.

	Montreal Healthy controls		Montreal Patient Controls		Montreal Suicide Attempters		F/χ²	P	Post-Hoc
	n = 38		n = 20		n = 16				
Gender, N males (%)	18	(47.3)	6	(30.0)	3	(18.8)	4.5	n.s.	
Age, mean (SD)	33.1	(8.2)	40.7	(10.3)	37.8	(10.5)	4.7	0.02	PC > HC
BDI score, mean (SD)	1.7	(1.7)	30.1	(11.9)	30.5	(11.5)	101.8	<10^−3^	PC, SA > HC
HDRS score, mean (SD)	1.2	(1.7)	30.1	(5.5)	27.3	(9.4)	216.9	<10^−3^	PC, SA > HC
Age at first depression (SD)	—	—	38.2	(11.3)	29.4	(9.9)	5.0	0.03	SA < PC
Number of depressive episodes (SD)	—	—	2.0	(1.2)	2.1	(1.1)	1.6	n.s.	
Family history of suicidal act, N (%)	—	—	5	(25)	3	(18.8)	1.9	n.s.	
Number of suicidal act (SD)	—	—	—	—	1.4	(1.3)	—	—	
History of violent suicidal act, N (%)	—	—	—	—	2	(13.3)	—	—	
SIS score (SD)	—	—	—	—	18.2	(5.7)	—	—	
History of physical or sexual childhood trauma, N (%)	—	—	4	(20)	7	(43.8)	3.0	n.s.	
	**Jena** **Healthy controls**		**Jena** **Patient Controls**		**Jena Suicide Attempters**		**F/χ²**	**P**	**Post-Hoc**
	**n = 28**		**n = 23**		**n = 26**				
Gender, N males (%)	9	(32.1)	4	(17.4)	7	(26.9)	1.5	n.s.	
Age, mean (SD)	36.7	(9.0)	35.1	(11.3)	36.8	(11.1)	0.2	n.s.	
BDI score, mean (SD)	1.9	(2.2)	31.3	(8.1)	25.8	(13.1)	81.4	<10^−3^	PC, SA > HC
HDRS score, mean (SD)	—	—	21.5	(9.4)	21.7	(10.4)	0.003	n.s.	—
Age at first depression (SD)	—	—	29.2	(12.6)	28.3	(10.3)	0.08	n.s.	
Number of depressive episodes (SD)	—	—	1.0	(0.9)	2.0	(2.2)	4.0	0.05	
Family history of suicidal act, N (%)	—	—	5	(21.7)	5	(19.2)			
Number of suicidal act (SD)	—	—	—	—	0.3	(0.7)			
History of violent suicidal act, N (%)	—	—	—	—	10	(38.5)			
SIS score (SD)	—	—	—	—	20.3	(4.2)			
Antidepressant medication							1.8	n.s.	
SSRI			10		10				
SNRI			10		9				
SSRI + Quetiapine			0		6				
unmedicated			3		1				
	**Montreal** **Healthy controls**		**Montreal** **Patient relatives**		**Montreal** **Suicide relatives**		**F/χ²**	**P**	**Post-Hoc**
	**n = 38**		**n = 16**		**n = 16**				
Gender, N males (%)	18	(47.3)	7	(43.8)	8	(50.0)	0.3	n.s.	
Age, mean (SD)	33.1	(8.2)	37.9	(8.7)	50.8	(9.2)	14.8	<10^−3^	SR > PR, HC
BDI score, mean (SD)	1.7	(1.7)	1.7	(2.2)	1.9	(3.1)	0.4	n.s.	
HDRS score, mean (SD)	1.2	(1.7)	1.8	(2.3)	2.3	(2.0)	2.1	n.s.	

*Footnotes*: HC: Healthy Controls; PC: Patient Controls; SA: Suicide Attempters; SR: Suicide Relatives; PR: Patient Relatives; SD: Standard Deviation; n.s.: non significant; BDI: Beck Depression Inventory; HDRS: Hamilton Depression Rating Scale; SIS: Beck Suicide Intent Scale; SSRI: Selective Serotonin Receptor Inhibitor; SNRI: Serotonin and Noradrenalin Receptor Inhibitor.

We applied the same inclusion and exclusion criteria as in our previous study^37^. Suicide attempts were defined as any acts carried out with some intent to die and thus did not include non-suicidal self-injuries. Exclusion criteria comprised a lifetime history of schizophrenia or bipolar disorder, a history of alcohol/substance abuse or dependence spanning the previous 6 months, a major general medical condition requiring ongoing pharmacological treatment, a lifetime history of severe head trauma or central nervous system disorder, and contraindication for MRI. All participants were right-handed and were English- or French-speaking natives in Montreal, and German-speaking natives in Jena. Informed written consent was obtained from all participants prior to their participation. This study was approved by the local ethics committees at the Douglas Mental Health University Institute in Montréal, Canada and at the University Hospital in Jena, Germany. The study protocol was carried out in accordance with the guidelines and regulations of the local ethics committees. Participants received a total of 100 Canadian dollars, or 10 euros per hour of participation.

#### Suicide relatives sample

As previously detailed^13,37^, two groups of non-depressed participants were recruited in Montreal: (1) 16 first-degree biological relatives of individuals who died from suicide (*Suicide relatives*); these suicide relatives had no personal history of suicide attempt. (2) 16 first-degree biological relatives of depressed patients (*Patient relatives*) with no personal or family (up to second biological degree) history of suicidal acts. The relatives of participants had suffered from MDD, but not schizophrenia or bipolar disorder. All participants had to be normothymic at time of participation and free of psychotropic medication for the last 6 months.

### Image acquisition

All scans in Montreal and Jena were acquired using a Siemens Magnetom Trio (Tim System 3 T) MRI scanner with a 12-channel (Montreal) or 64-channel (Jena) head coil. Participants were instructed to keep their eyes closed throughout scanning. In Montreal, T2*-weighted images were obtained using a gradient-echo EPI sequence (TR = 2090 ms, TE = 30 ms, flip angle 90°) with 38 contiguous transverse slices of 3.5 mm thickness and an in-plane resolution of 3.5 × 3.5 mm². A series of 285 whole-brain volume sets was acquired in one session. In Jena, T2*-weighted images were obtained using a gradient-echo EPI sequence (TR = 2520 ms, TE = 30 ms, flip angle 90°) with 45 contiguous transverse slices of 2.5 mm thickness and an in-plane resolution of 2.5 × 2.5 mm² covering the entire brain. A series of 240 whole-brain volume sets were acquired in one session. High-resolution, whole-brain T1-weighted acquisitions were collected using a magnetization prepared rapid gradient echo (MPRAGE) sequence with 1 mm³ isotropic voxels in both Montreal and Jena. All scans were visually checked for motion artefacts. A neuroradiologist inspected the T1-weighted images for gross pathological findings in Jena and confirmed their absence.

### Preprocessing of the rs-fMRI data

Standard preprocessing was performed using AFNI (http://afni.nimh.nih.gov/afni/) and FSL (www.fmrib.ox.ac.uk/fsl). The first five images were discarded to obtain steady-state tissue magnetization. Preprocessing included slice timing correction and realignment to the first volume using a rigid body transformation. To check for potential differences in motion between groups, we calculated the mean absolute displacement of each brain volume from the translation parameters in the x, y, and z directions as well as the mean scan-to-scan displacement in 3D space, as described previously^38^. No significant differences in these parameters were detected; no significant correlations between motion and topological parameters could be found. Afterwards, extra-cerebral tissue was removed from the anatomical images using the Robust Brain Extraction (ROBEX) tool^39^. The skull-stripped brains were aligned with the standard Montreal Neurological Institute (MNI) brain. A within-subject registration was then performed between functional and anatomical images. Finally, functional images were spatially normalized to the MNI space by applying normalization parameters. Due to different voxel resolutions, the Montreal data were smoothed using a Gaussian filter of 7 mm FWHM and the Jena data using a Gaussian filter of 5 mm FWHM. Further additional preprocessing steps were (i) removal of linear and quadratic trends and of several sources of variance, i.e. head-motion parameter, cerebrospinal fluid and white matter signals, as well as (ii) temporal band-pass filtering, retaining frequencies in the 0.01–0.08 Hz band. For the whole-brain network analysis, 262 independent anatomical regions of interest (ROI) were defined based on the coordinates from the extensively validated parcellation system provided by Power *et al*.^40^ (Table S5).

### Statistical analysis: graph theoretical analysis

Using the Brain Connectivity Toolbox^41^, the between-group differences in the topologic properties of the functional connectome were assessed for functional segregation (clustering coefficients), functional integration (global efficiency), and resilience (assortativity) of networks. We additionally calculated rich-club coefficients, which describes to what extent high-degree nodes (“hubs”) are more tightly connected among themselves than nodes of a lower degree^42^. Rich club coefficients are assumed to reflect network resilience and the efficiency of global information flow^30^.

Individual correlation matrices were derived, extracting the mean time series from the 262 ROIs. We focused on differences in positive correlations in each individual connectivity matrix for the analysis of global topological parameters. We used the average positive matrix from the healthy group with a network density of 45% (corresponding to 15403 edges). Furthermore, short-distance (less than 20 mm) correlations were discarded following Power *et al*.^40^ to avoid possible shared signals between nearby nodes.

In order to circumvent a potential problem in applying one arbitrary threshold, potential between-group differences in global topological network metrics were compared using a permutation-based, two-sample and two-tailed t-test for each network density between 10% and 34%, in increments of 1%, as also used by Zhang *et al*.^25^. All statistical comparisons were controlled for age, gender and site (including the factor medication).

**Figure 1:**
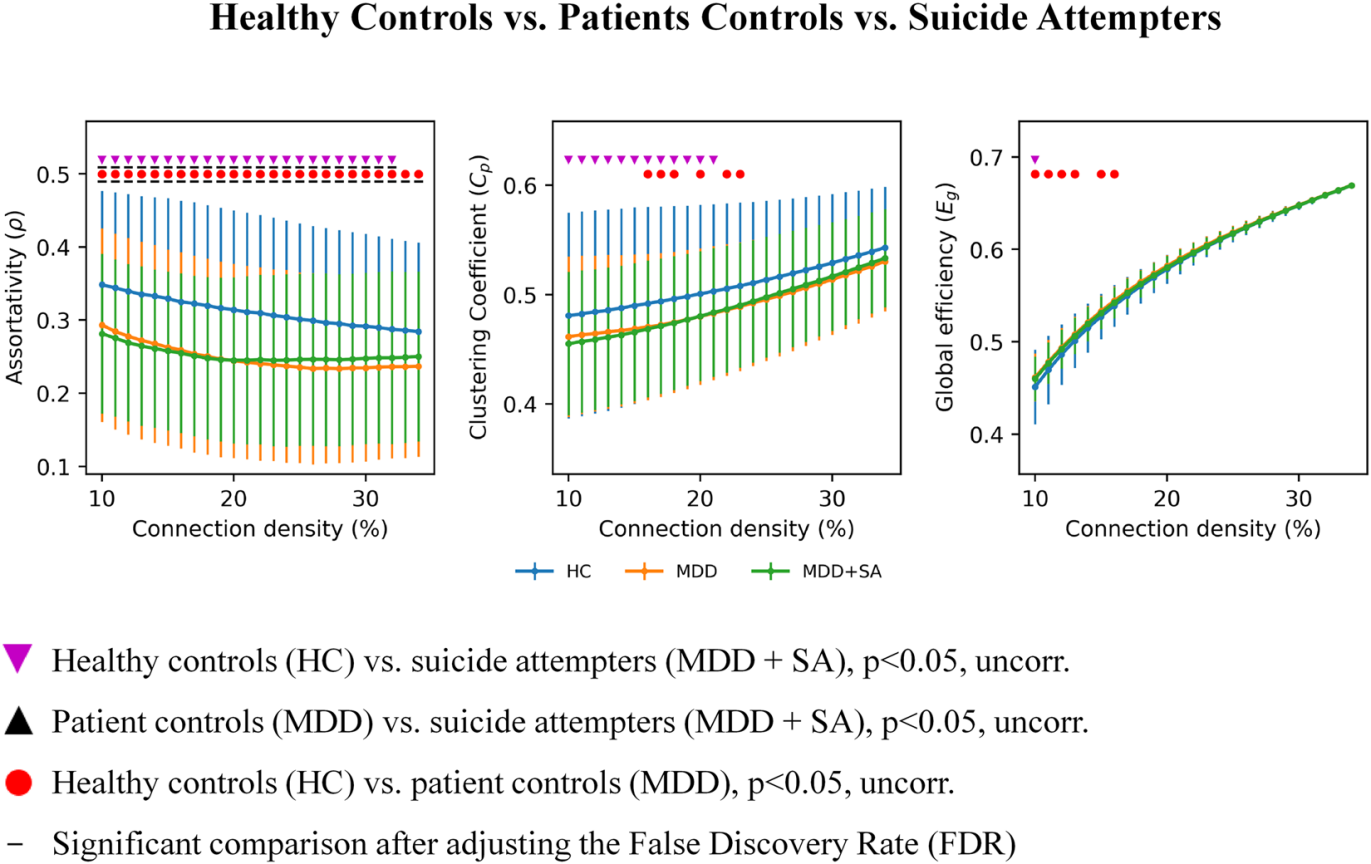
Significant differences in graph topological measures (assortativity, clustering coefficients, global efficiency) are illustrated between suicide attempters, patient controls and healthy controls.

**Figure 2:**
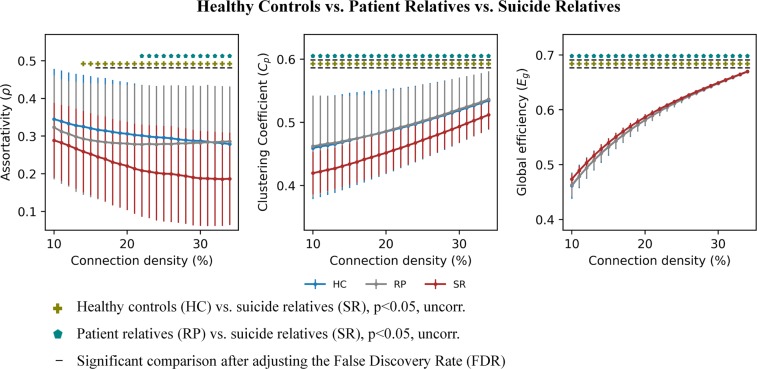
Significant differences in graph topological measures (assortativity, clustering coefficient, global efficiency) are illustrated between healthy relatives of suicide victims, healthy relatives of depressed patients with no family histories of suicidal behavior, and healthy controls.

Moreover, since there is no formal consensus regarding the selection of a single sparsity threshold, we thus presented statistical comparisons over a range of sparsity thresholds (S), thus increasing the alpha error probability. However, presenting comparisons for one network density only, can be considered as arbitrary. To account for the problem of multiple comparisons and of using one arbitrary sparsity, we indicated in Figs 1 and 2 comparisons surviving the adjusted false discovery rate (FDR) according to the Benjamini-Hochberg procedure^43^ in addition to statistical comparisons, which survived the uncorrected threshold of p < 0.05.

The rich-club coefficient Φ_(k)_ at each level of degree, k, was computed as the ratio of the number of connections between nodes within the *k*th subgraph and the total number of possible connections between them based on group averaged networks^44,45^. The network density was set to include the top 7% of the strongest positive connections to reduce the number of potentially spurious connections. A similar network density was used in previous studies investigating rich-club organization^46,47^. We also tested the stability of rich-club organization across a range of different network densities varying from 7% to 15%, which showed a stable behavior. The rich-club coefficient was additionally compared and normalized to sets of “equivalent” random networks Φ_norm(k)_. We generated a thousand random networks having equal size and degree distributions. The network is considered to have rich-club organization, when Φ_norm(k)_ is greater than 1 for a continuous range of k^44^. Permutation tests were used to compute the significance of the rich-club curves. The generated one thousand random networks produced a null distribution of rich-club coefficients (Φ_rand(k)_). Using this distribution, a p-value was assigned to Φ_norm(k)_ as the percentage of random (null) values that exceeded Φ_rand(k)_ (p < 0.05, one-tailed, Bonferroni corrected). Differences in the rich-club organization between suicide attempters, patient controls, relatives and healthy controls were also tested for significance. For each degree k of the random network of one group R_group1(k)_ and the random network of the other group R_group2(k)_, the difference between the rich-club coefficients for R_group1(k)_ and for R_group2(k)_ produced a null distribution of a thousand random differences. Using this distribution, a p-value was assigned to each observed difference Φ_group1(k)–_Φ_group2(k)_ (p < 0.05, two-tailed, FDR corrected).

### Statistical analysis: network-based statistic (NBS)

Group differences between FC matrices were examined using the framework of the NBS introduced by Zalesky *et al*.^33^. NBS is a validated non-parametric method to avoid the multiple comparison problems due to mass univariate significance testing in network connectivity. The NBS analysis followed a procedure used in our previous study^32^. The first step in the analysis was the calculation of the connectivity matrices for each group. The potentially confounding effects of age, gender and site (including the factor medication) was (as for the between comparison of topological parameters) controlled by regressing out these variables from the individual connectivity matrices. Negative edges were removed from the individual subject’s connectivity matrix by masking it with the positive average matrix from the healthy subjects, as the reference connectivity matrix. This was done because all subjects must have the same network to be able to perform statistical comparisons of the FC differences. Secondly, a primary component-forming threshold (with a threshold of p < 0.005) was applied to identify all edges displaying potential differences in connectivity strength. Thirdly, all sub-threshold edges were assessed for mutual connections forming topological clusters that may point towards the existence of non-chance clusters. Permutation testing was then applied to compute p-values for every component previously identified^32^. To this end, the first three steps were repeated for each of the 10,000 random permutations of group assignments (i.e. patients or healthy controls or relatives), while noting the maximum cluster sizes of components resulting in a null distribution for the largest component size^32^.

## Results

### Graph analyses

#### Suicide attempters sample

A significant reduction (p < 0.05, FDR corr.) in assortativity was detected between the total sample of suicide attempters and healthy controls as well as between patient controls and healthy controls nearly across all studied network densities within the small-world regime (Fig. 1). Additionally, at the uncorrected threshold level (p < 0.05), lower clustering coefficients were found in suicide attempters compared to healthy controls across the first 12 network densities, and in patient controls over 6 midrange network densities. Finally, higher global efficiency was detected in suicide attempters compared to healthy controls at the network density of 10% as well as in patient controls compared to healthy controls at the six first network densities (again, however, only at the uncorrected threshold). There were no significant differences in these three global parameters between suicide attempters and patient controls.

When the two sites were examined separately and also controlled for age and gender, patient control groups showed different profiles in Jena and Montreal while both suicide attempters and healthy controls were more similar between sites (Fig. S1A,B).

#### Suicide relatives sample

Suicide relatives exhibited significant differences (p < 0.05, FDR corr.) compared to healthy controls in all three topological measures across a wide range of network densities (Fig. 2). Compared to patient relatives, suicide relatives exhibited significantly lower assortativity at the uncorrected threshold, and significantly lower clustering coefficient and higher global efficiency at the FDR adjusted threshold. There was no significant difference between patient relatives and healthy controls.

### Rich club analysis

#### Suicide attempters sample

**Figure 3:**
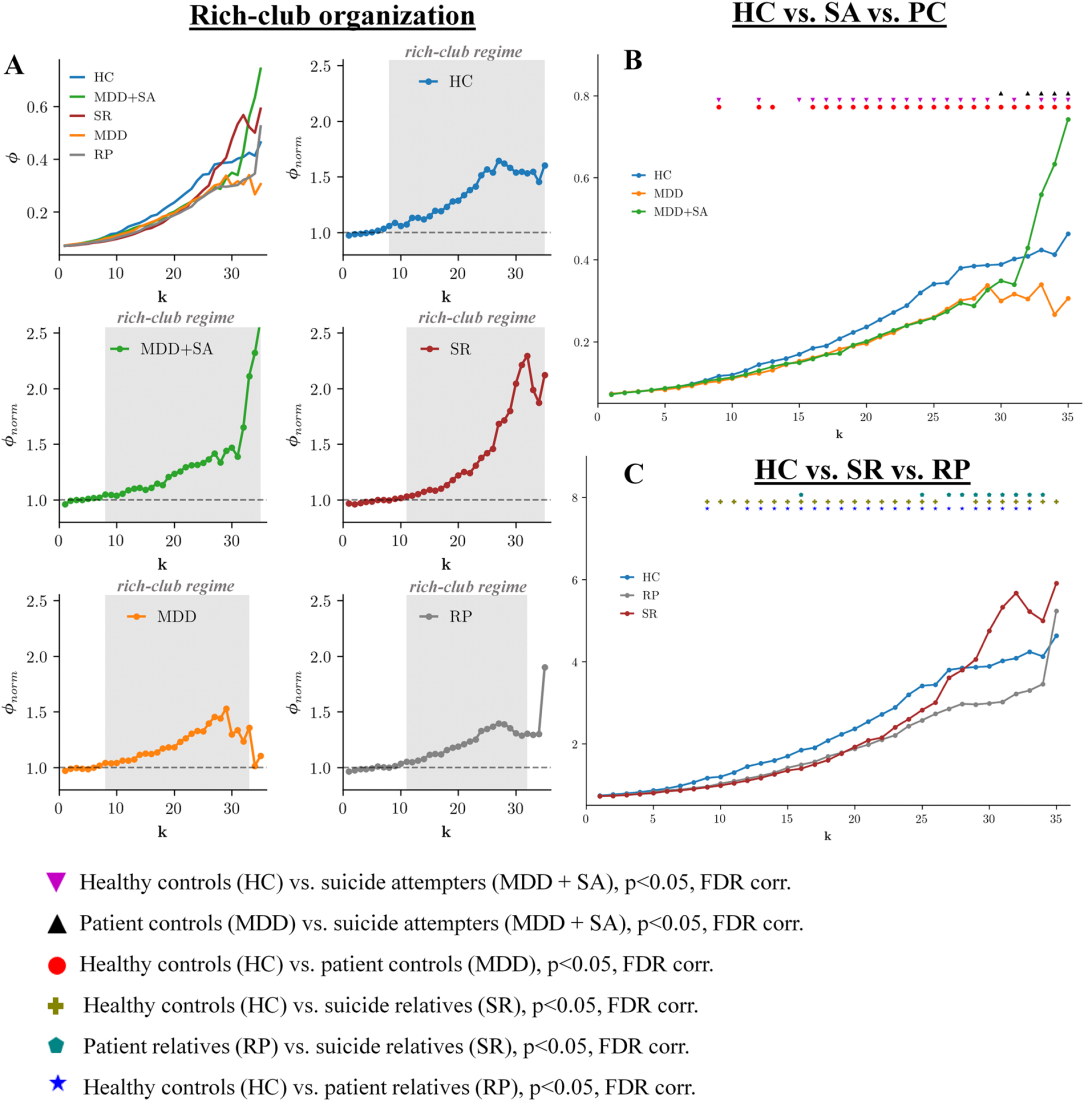
Rich-club organization and between group differences in rich-club coefficients for patients and relatives. The (**A**) shows the rich-club coefficient values Φ_(k)_ (k is the degree of a node) and group-specific normalized rich-club coefficient Φ_norm(k)_ curves for the group-averaged brain network. The rich-club regime is indicated by the dark gray area, which is defined by a significant difference of Φ_norm(k)_ from the random null distribution (permutation test, p = 0.05, Bonferroni corrected). (**B,C**) show significant between-group differences (as indicated by the corresponding symbols), computed for each k (permutation test). All depicted significant comparisons survived the adjusted false discovery rate (FDR) of p = 0.05.

We found significant rich-club organization (rich-club regime) in the functional connectome of studied groups across several levels of *k* (indicated by the dark gray area in Fig. 3A). (Fig. 3A,B) Furthermore, we observed significantly lower coefficients across several low levels of k within the rich-club regime in suicide attempters and patient controls than in healthy controls (Fig. 3B). However, a sharp rise was observed in the normalized rich-club coefficient of suicide attempters at around k = 30 with significantly higher rich-club coefficients in this group compared to patient controls and healthy controls (while patient controls remained at lower level than healthy controls) indicating the presence of densely interconnected high-degree nodes.

#### Suicide relatives sample

A similar profile was found in suicide relatives as in suicide attempters, with significantly lower coefficients across several low levels of *k* in suicide relatives and patient relatives than in healthy controls, and significantly higher rich-club coefficients in this group compared to patient relatives and healthy controls (while patient relatives remained at lower levels compared to healthy controls) at around k = 30. (Fig. 3C).

### Correlation analysis

In the total sample of patients and healthy controls, there were no significant associations between topological metrics across different network densities and age or gender. Furthermore, no significant associations could be found between depression severity as indicated by HDRS and BDI total scores and topological metrics across different network densities.

### Network-Based Statistics (NBS) of FC

#### Suicide attempter sample

**Figure 4:**
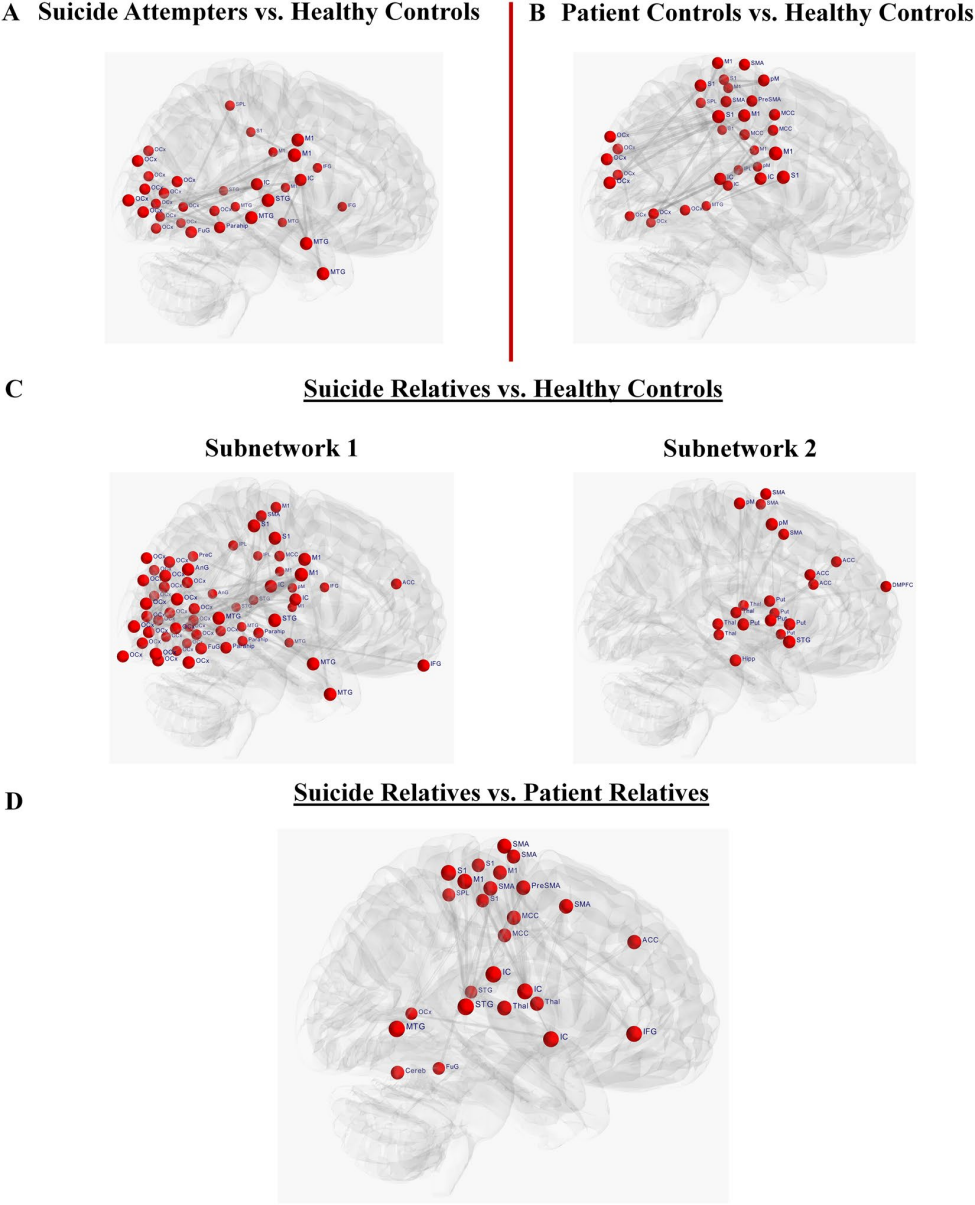
Group comparisons in functional connectivity matrices using Network-Based statistics (NBS). Significant group differences between functional connectivity (FC) matrices using the framework of the network-based statistic (NBS) introduced by Zalesky *et al*.^33^ are illustrated. NBS is a validated nonparametric method to avoid the multiple comparison problems due to mass univariate significance testing in FC. (**A**) NBS analysis revealed a single network of decreased FC in suicide attempters as compared with healthy controls (p = 0.04, FWER) comprising a total of 33 nodes connected by 34 edges and including occipital regions (OCx), right fusiform gyrus (FuG), middle (MTG) and superior temporal gyrus (STG), left inferior frontal gyrus (IFG), right posterior insula (IC), bilateral primary motor (M1) and left somatosensory (S1) cortices, left superior parietal lobe (SPL), and right parahippocampal gyrus (Parahip). (**B**) NBS analysis revealed a single network of decreased FC in patient controls as compared with healthy controls (p = 0.03, FWER) comprising a total of 33 nodes connected by 39 edges and including several nodes located in the somatosensory-motor (M1 and S1) and occipital regions, midcingulate cortex (MCC), posterior IC, left MTG, and inferior parietal lobe (IPL) and SPL. (**C**) NBS analysis revealed two subnetworks of decreased FC in relatives of suicide victims as compared with healthy controls. The first subnetwork (p = 0.001, FWER) comprised a total of 61 nodes connected by 118 edges and included several occipital, temporal and somatosensory-motor regions, bilateral IFG, parahippocampal gyrus, right posterior IC, left IPL, bilateral angular gyrus (AnG), and precuneus (PreC). The second subnetwork (p = 0.02, FWER) comprised a total of 21 nodes connected by 26 edges and included bilateral putamen (Put), bilateral anterior cingulate cortex (ACC), dorsomedial prefrontal cortex (DMPFC), bilateral supplementary motor area (SMA), right premotor cortex (pM), bilateral thalamus (Thal), right STG, and right hippocampus (Hipp). (**D**) NBS analysis revealed a single network of decreased FC in relatives of suicide victims as compared with relatives of patients with no family history of suicidal behavior (p = 0.02, FWER) comprising a total of 26 nodes connected by 28 edges and including somatosensory-motor regions, ACC and MCC, right IFG, right posterior IC, bilateral thalamus, bilateral STG, and right MTG, left SPL, left fusiform gyrus and middle occipital gyrus (OCx).

NBS analysis revealed a single network of decreased FC in suicide attempters as compared with healthy controls (p = 0.04, FWER) (Fig. 4A,B). The network (Fig. 4A and Table S1) comprised a total of 33 nodes connected by 34 edges, and consisted of nodes located in the occipital regions, middle and superior temporal gyrus, left inferior frontal gyrus, right posterior insula, bilateral primary motor (M1) and left somatosensory (S1) cortices, the left superior parietal lobe, and right parahippocampal gyrus.

Comparing patient controls and healthy controls, significant (p = 0.03, FWER) differences in FC were detected in a network of 33 nodes connected by 39 edges (Fig. 4B and Table S2). This network included several nodes located in the somatosensory-motor and occipital regions, midcingulate cortex, posterior insula, left middle temporal gyrus, and inferior and superior parietal lobe.

No significant differences were found between suicide attempters and patient controls.

#### Suicide relatives sample

Suicide relatives exhibited two sub-networks (components) of decreased FC compared with healthy subjects (Fig. 4C and Table S3) (Fig. 4C,D). The first significantly different network (p = 0.001, FWER) comprised a total of 61 nodes connected by 118 edges lying in several occipital, temporal and somatosensory-motor regions. Further nodes were located in bilateral inferior frontal gyrus, parahippocampal gyrus, right posterior insula, left inferior parietal lobe, bilateral angular gyrus, and precuneus.

The second significantly different network (p = 0.02, FWER) was composed of 21 nodes connected by 26 edges mainly located in the fronto-cingulo-striatal network, i.e. in the bilateral putamen, bilateral anterior cingulate cortex, dorsomedial prefrontal cortex, bilateral supplementary motor area, right premotor cortex, bilateral thalamus, right superior temporal gyrus, and right hippocampus. There were no significant differences in the opposite direction as well as between patient relatives and healthy controls.

Comparing suicide relatives to patient relatives (p = 0.02, FWER), NBS analyses revealed a single network of decreased FC in suicide relatives (Fig. 4D and Table S4). The network comprised a total of 26 nodes connected by 28 edges and included somatosensory-motor regions, anterior and middle cingulate cortex, right inferior frontal gyrus, right posterior insula, bilateral thalamus, bilateral superior temporal gyrus, and right middle temporal gyrus, left superior parietal lobe, left fusiform gyrus and middle occipital gyrus. There were no significant differences in the opposite direction.

## Discussion

The normal functioning of complex processes such as cognition, emotion or social interactions requires precisely orchestrated interactions within and between specific neural networks. Aberrant topological attributes and connectivity patterns have been found in several psychiatric disorders^25,31,32^, suggesting that abnormal brain network functioning underlies complex psychiatric disorders. In the present study, we investigated whether suicidal behavior, which is associated with persisting abnormal cognitive and affective processes as well as difficulties in social interactions^7^, is associated with a functional disorganization of brain networks at both the global and local scales. To meet this aim and in order to increase the sample size to have a higher statistical power, we combined resting state data from patients with MDD with and without a history of suicide attempt from Montreal, Canada and from Jena, Germany. We further recruited one sample of healthy relatives of suicide victims in order to investigate the most robust heritable functional patterns.

Regarding our first hypothesis in patients, we had to reject this hypothesis for most investigated parameters. We could not differentiate patients with vs. without a personal history of suicide attempt based on the three investigated global topological parameters (i.e. assortativity, clustering coefficient and global efficiency) as well as using local FC based on NBS. However, both patient groups showed significant differences in these parameters compared to healthy controls suggesting a main effect of depression. Nevertheless, the analysis of the rich-club organization significantly differentiated suicide attempters from patient controls with respect to high-degree nodes and, surprisingly, indicated higher rich-club organization in suicide attempters. Regarding our second hypothesis in relatives, findings were clearer, and the initial hypothesis was confirmed. We could differentiate healthy relatives of suicide victims from healthy relatives of MDD patients without suicide attempt and healthy controls in terms of the three global parameters, local FC, and rich-club-organization. Importantly, changes in measurements in our groups of interest - suicide attempters and suicide relatives - were always in the same direction.

The difficulty in differentiating suicide attempters from patient controls (but not suicide relatives from patient relatives) may be explained by the heterogeneity of patients with MDD. First, our own datasets (Fig. S1) show a large variability in topological measures between patient control groups from Montreal and Jena. Potentially different neurophysiological biotypes were recruited in this group in the present study^48^. Some differing sample characteristics regarding medication status or inpatient treatment may also contribute to between-site differences. Moreover, previous studies investigating functional connectomics in MDD patients produced mixed results pointing toward potential heterogeneity with regard to depression-associated alterations. For example, Zhang *et al*.^25^ observed higher global efficiency, which suggests a shift toward randomization in brain networks. In contrast, Meng *et al*.^49^ reported decreased global efficiency in depressed patients. This inconsistency might be related to specific differences in patient characteristics. For instance, Zhang *et al*.^25^ included drug-naive and first-episode patients, whereas Meng *et al*.^49^ studied medicated patients with different types of antidepressants and with multiple depressive episodes. An additional rs-fMRI study by Lord *et al*.^50^ with medicated MDD patients reported no significant differences in these global measures compared to healthy controls. Thus, these studies indicate putative effects of patient-specific characteristics on the functional connectome. Interestingly, an MRI study^37^ recently showed differences in structural volumes according to the suicidal means used (violent vs. medication), highlighting potentially contributing factors of heterogeneity among suicide attempters. In contrast to patients, a close familial history of suicide death may yield higher group homogeneity with respect to abnormal global network parameters. Fornito *et al*.^51^ showed in a twin study that 60% of the individual variance in some cost-efficiency topological metrics could be explained by additive genetic effects. In the structural MRI study mentioned above, significant differences were also found between suicide attempters with and without a family history of suicidal behavior^37^. Hence, the present results might suggest that genetic factors potentially contribute to the global network changes and altered connectivity observed in suicidal behavior.

A first set of main findings concerns the global brain functional organization. The most consistent result across the three investigated topological measurements in patients was found with respect to assortativity, a measure of network resilience. Significantly reduced assortativity was detected nearly across all network densities in suicide attempters compared to healthy controls. Moreover, significantly and markedly reduced assortativity as well as reduced clustering coefficient and increased global efficiency was observed in relatives of suicide victims when compared to patient relatives and healthy controls. The vulnerability to suicidal behavior therefore appears to be associated with reduced assortativity and impaired segregation in brain functional organization. The organization of the human connectome has been shown to have an optimal balance between segregation and integration to enable efficient processing of external and internal stimuli^52^. Interestingly, while suicide attempters showed only few abnormalities in the integration or segregation parameters (only at the uncorrected statistical threshold), suicide relatives showed increased global efficiency vs. both control groups, i.e. markers of increased integration. This raises the question if increased integration represents a compensatory mechanism to poor network resilience and segregation, and subsequently a protective factor against a suicidal act in this particular population.

Assortativity is defined as the Pearson correlation coefficient of the node degrees of connected pairs of edges^29,44^. Nodes of similar degree (i.e. similar number of connections) tend to be connected to each other in networks that exhibit high assortativity. In such networks, high degree nodes, commonly-called hubs, are likely to be connected to each other, which makes the network more resilient against selective node failure^29^. Our findings of decreased assortativity in suicide attempters and (even more strongly) in suicide relatives suggest that the global brain functional network in these groups may be less resilient to “assaults”. Suicidal behavior has been modeled as the potential complex outcome of stressful events, such as interpersonal conflicts, affecting vulnerable individuals, with some of the vulnerability factors being heritable^53,54^. Low assortativity may therefore be a general and heritable factor of neural vulnerability. In individuals with low assortativity, stressors may cause a particular detrimental effect on the global network functioning. To the best of our knowledge, no study has previously reported impaired assortativity in the functional connectome in relation to suicidal behavior. This result will therefore have to be replicated.

In order to pursue the investigation of resilience among our groups of interest, we additionally measured the rich-club organization. The rich-club coefficient measures the density of connectivity between high-degree nodes^44^. It takes a central position in the brain’s network topology and describes the phenomenon that nodes with high degrees tends to interconnect with themselves, providing important information about higher-level network topology with respect to the integration of information among different neural subsystems^30^. Previous studies indicated abnormally reduced rich-club organization in patients with schizophrenia^31^ and unaffected siblings^55^, in patients with bipolar disorder^56^ and patients with Alzheimer’s disease^57^.

In line with findings of reduced assortativity, we found that depressed patients and relatives exhibited a significantly lower rich-club coefficient then healthy controls across most degrees. However, regarding the highest degree nodes, a sharp rise in the normalized rich-club coefficient was detected at around k = 30 in suicide attempters and suicide relatives only. These results suggest the presence of an imbalance in rich-club organization in relation to suicidal behavior. Due to their central position in the topology of the network, connections between these rich club nodes seem to have a central role in the efficient integration of information from distant brain regions^30^. Misic *et al*.^58^ demonstrated on macaque brains that the rich-club nodes mediate most of the information flow. Therefore, damage to rich-club connections may impact the network efficiency more severely than random damage to the network. Supporting this notion, robustness analyses showed that a disruption of high-degree node connections is associated with a marked reduction in the topological integration of the network compared to random removal of other edges^30^. Thus, such disorganization in the so called “rich-club” may contribute to the vulnerability inherent to suicidal behavior. This will have to be further explored.

A second set of main results relates to the brain organization. In patients, NBS analyses revealed very similar decreased FC alterations in a network comprising somatosensory-motor, insula, superior parietal, and occipital regions in both suicide attempters and patient control groups vs. healthy controls. Further nodes were found in the inferior frontal, parahippocampal, fusiform and superior temporal gyri in suicide attempters only, and in additional somatosensory-motor regions and middle cingulate cortex in patient controls only. There was no significant difference between suicide attempters and patient controls. Again, heterogeneity among patient groups may partly explain this latter finding. Moreover, most identified regions may be mainly related to depression more than to more specific suicide vulnerability *per se*.

In contrast to suicide attempters, suicide relatives showed significantly lower FC in comparison to both patient relatives and healthy controls. Compared to healthy controls, two sub-networks could be identified. The first sub-network comprised a number of occipital, somatosensory-motor regions as well as some temporal, insular, frontal and anterior cingulate regions. Thus, the central and posterior parts of this network are largely overlapping with the networks found in depressed patients. The second sub-network consisted of the dorsomedial prefrontal cortex, the anterior cingulate, the striatum, and the thalamus. In comparison to patient relatives, suicide relatives exhibited lower FC in a network including mainly the anterior cingulate, inferior frontal gyrus, thalamus, insula, somatosensory-motor regions, superior and inferior parietal regions, superior temporal gyrus, and the cerebellum.

Overall, this complex combination of results mainly points toward an association between the vulnerability to suicidal behavior and reduced FC within a large network comprising the ventral and dorsal prefrontal cortex, the anterior cingulate, thalamus, striatum, and possibly the insula, fusiform gyrus and cerebellum. The implication of other regions (parahippocampal gyrus, some somatosensory and motor regions, middle temporal gyrus) is more ambiguous. Proper functioning of this network is crucial for successful cognitive control as well as successful inhibition of pre-potent motor responses^59^. Most regions reported here have previously been associated with suicidal behavior in fMRI studies. Altered responses in the ventrolateral, dorsolateral, dorsomedial prefrontal cortices and striatum have been associated with impaired decision-making, risk processing and impulsivity in suicide attempters^9,10,54^ and suicide relatives^13^, and higher sensitivity to social disapproval in suicide attempters^12,54^. Structural studies investigating white matter integrity further lend support to altered connectivity between prefrontal and subcortical regions in suicide attempters^60–62^. The precise role of the cerebellum in suicidal behavior has to be determined in future studies.

Several limitations of the study have to be underlined. First, samples were of limited size, notably the relative sample. While this is a complicated sample to recruit, more studies with this population will be necessary. The effect of psychopharmacological medication on the functional connectome should also be better understood to explain differences between Montreal and Jena samples, mainly regarding depressed patient control groups. Furthermore, a classification analysis on the global parameters might be helpful to differentiate suicide attempters from patient controls or healthy controls. However, as shown in a recent study^63^, a sufficient amount of data are required to generate a reliable model in order to have a good estimate of the prediction accuracy. This was not possible here due to the sample size. It will be important to investigate the structural rich-club organization based on diffusion tensor imaging (DTI) data in suicide attempters and relatives of suicide victims as the anatomical substrate of the functional connectome.

In conclusion, the present study supports the hypothesis that deficits in functioning and organization of brain networks possibly contribute to the risk of suicidal behavior. More specifically, reduced resilience, abnormal functional segregation and integration of the whole brain network, as well as decreased FC at rest in a large network of identified brain regions may be accounted to heritable mechanisms associated with the vulnerability to suicidal acts. Any intervention facilitating the functional re-organization of the system may help to diminish the long-term suicidal risk. Moreover, the potential utility of the topological metrics as predictive markers of suicidal behavior will have to be explored in classification studies as well as in prospective studies.

## Supplementary information

Supplementary material
