# Supplementary material for: Connectomics-Based Functional Network Alterations in both Depressed Patients with Suicidal Behavior and Healthy Relatives of Suicide Victims
Source: Sci Rep. 2019 Oct 4;9:14330. doi: 10.1038/s41598-019-50881-y (PMC6778100; doi:10.1038/s41598-019-50881-y)
Supplement: Supplementary file 1 — Supplementary material [file 41598_2019_50881_MOESM1_ESM.pdf]

Connectomics-Based Functional Network Alterations in both Depressed Patients with Suicidal Behavior and Healthy Relatives of Suicide Victims.

Gerd Wagner PhD [1], Feliberto de la Cruz MSc [1], Stefanie Köhler MSc [1], Fabricio Pereira PhD [4], Stéphane Richard-Devantoy MD PhD [2], Gustavo Turecki MD PhD [2], Karl-Jürgen Bär MD [1+], Fabrice Jollant MD PhD [2,3+].

[1] Department of Psychiatry and Psychotherapy, Jena University Hospital, Philosophenweg 3, 07743 Jena, Germany;

[2] McGill group for Suicide Studies, McGill University & Douglas Mental Health University Institute, Montréal, Canada;

[3] Université Paris-Descartes, Faculté de médicine & Clinique des Maladies Mentales et de l'Encéphale (CMME), Hôpital Sainte-Anne, Paris, France;

[4] Departments of Radiology & Psychiatry, University Hospital Center of Nîmes, Nîmes, France.

+ Both authors contributed equally to this work

Corresponding author:

Gerd Wagner, PhD

Department of Psychiatry and Psychotherapy, Jena University Hospital,

Jena, Germany

Philosophenweg 3

07743 Jena, Germany

Tel.: +49(0)36419390421

E-mail: wagner.gerd@uni-jena.de

Supplementary material

Figure S1: Significant differences in graph topological measures (assortativity, clustering coefficients, global efficiency) are illustrated between suicide attempters, patient controls, and healthy controls for Montreal (A) and Jena (B) samples separately, controlled for age and gender.

A **Montreal sample**

Healthy Controls vs. Patients Controls vs. Suicide Attempters

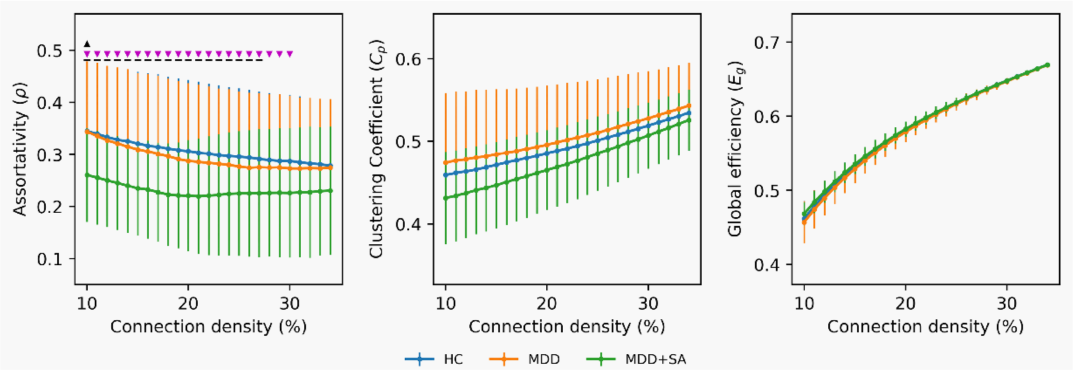

B **Jena sample**

Healthy Controls vs. Patients Controls vs. Suicide Attempters

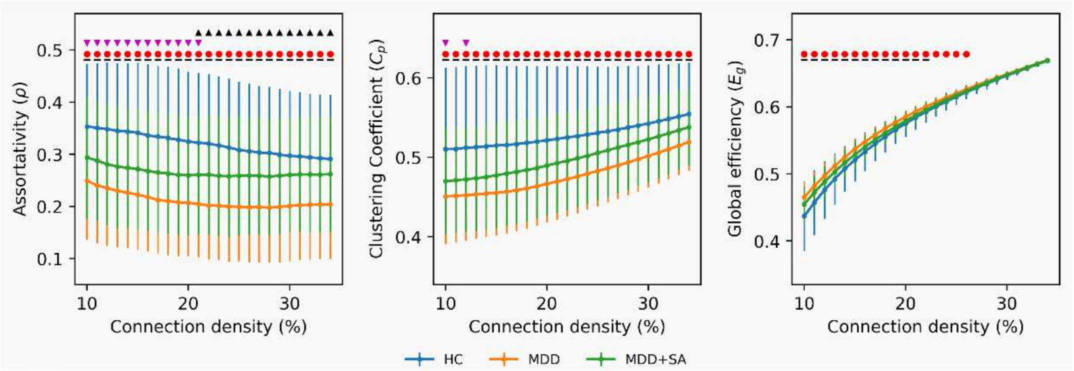

▼ Healthy controls (HC) vs. suicide attempters (MDD + SA), $p<0.05$, uncorr.

▲ Patient controls (MDD) vs. suicide attempters (MDD + SA), $p<0.05$, uncorr.

● Healthy controls (HC) vs. patient controls (MDD), $p<0.05$, uncorr.

– Significant comparison after adjusting the False Discovery Rate (FDR)

Table S1: MNI coordinates of significant group differences between healthy controls and suicide attempters are presented for the Network-Based Statistics (NBS) analyses at the whole-brain level.

Healthy controls vs. Suicide attempters, p = 0.04, FWER			
**Node**	**MNI coordinate**		
	**x**	**y**	**z**
Inferior frontal gyrus	-46	10	24
Inferior frontal gyrus	-50	26	0
Posterior insula	32	-26	12
Posterior insula	36	-10	14
Middle temporal gyrus	-50	-42	0
Middle temporal gyrus	52	-28	-4
Middle temporal gyrus	52	-2	-16
Middle temporal gyrus	52	6	-30
Middle temporal gyrus	-56	-12	-10
Superior temporal gyrus	-56	-50	10
Superior temporal gyrus	58	-16	8
Precentral gyrus	-50	-12	34
Precentral gyrus	-56	-10	12
Precentral gyrus	66	-8	24
Precentral gyrus	52	-6	32
Postcentral gyrus	-46	-32	46
Parahippocampal gyrus	18	-46	-10
Superior parietal lobe	-30	-44	60

Cuneus	-24	-92	18
Cuneus	8	-72	10
Cuneus	-14	-90	32
Cuneus	6	-82	6
Cuneus	-8	-80	8
Cuneus	24	-88	24
Lingual gyrus	20	-86	-2
Lingual gyrus	-16	-72	-8
Lingual gyrus	-16	-52	-2
Fusiform gyrus	26	-60	-10
Middle occipital gyrus	-42	-74	0
Middle occipital gyrus	-26	-90	4
Middle occipital gyrus	36	-82	2
Middle occipital gyrus	-46	-76	-10
Inferior occipital gyrus	-40	-88	-6

Table S2: MNI coordinates of significant group differences between healthy controls and patient controls are presented for the Network-Based Statistics (NBS) analyses at the whole-brain level.

**Healthy controls vs. Patient controls, p = 0.03, FWER**			
**Node**	**MNI coordinate**		
	**x**	**y**	**z**
Medial frontal gyrus	10	-18	74
Medial frontal gyrus	4	-18	58
Posterior insula	-30	-28	12
Posterior insula	32	-26	12
Posterior insula	36	-10	14
Middle temporal gyrus	-50	-42	0
Midcingulate cortex	10	-2	44
Midcingulate cortex	-10	-2	42
Midcingulate cortex	-14	-18	40
Precentral gyrus	20	-8	64
Precentral gyrus	-38	-28	70
Precentral gyrus	2	-28	60
Precentral gyrus	38	-18	44
Precentral gyrus	-50	-12	34
Precentral gyrus	-52	-10	24
Precentral gyrus	66	-8	24
Postcentral gyrus	-22	-30	72
Postcentral gyrus	14	-32	74
Postcentral gyrus	28	-40	60
Postcentral gyrus	-46	-32	46

Postcentral gyrus	50	-20	42
Postcentral gyrus	56	-6	14
Superior parietal lobe	-30	-44	60
Inferior parietal lobe	-52	-22	22
Cuneus	-24	-92	18
Cuneus	-14	-90	32
Cuneus	16	-88	36
Cuneus	24	-88	24
Lingual gyrus	-16	-72	-8
Lingual gyrus	-16	-52	-2
Middle occipital gyrus	36	-84	12
Middle occipital gyrus	-46	-76	-10
Inferior occipital gyrus	-40	-88	-6

Table S3: MNI coordinates of significant group differences between healthy controls and relatives of suicide victims are presented for the Network-Based Statistics (NBS) analyses at the whole-brain level.

Healthy controls vs. Relatives of suicide victims, 1st component, p < 0.001, FWER			
**Node**	**MNI coordinate**		
	**x**	**y**	**z**
Medial frontal gyrus	-6	-20	66
Medial frontal gyrus	2	-28	60
Inferior frontal gyrus	34	54	-18
Inferior frontal gyrus	-46	10	24
Anterior cingulate cortex	-8	48	24
Midcingulate cortex	-14	-18	40
Posterior insula	36	-10	14
Posterior insula	44	-22	20
Precentral gyrus	52	-6	32
Precentral gyrus	66	-8	24
Precentral gyrus	-56	-10	12
Precentral gyrus	-52	-10	24
Precentral gyrus	-50	-12	34
Postcentral gyrus	50	-20	42
Postcentral gyrus	48	-30	48
Superior temporal gyrus	58	-16	8
Superior temporal gyrus	-38	-34	16
Superior temporal gyrus	-56	-40	14
Middle temporal gyrus	52	6	-30

Middle temporal gyrus	52	-2	-16
Middle temporal gyrus	-56	-12	-10
Middle temporal gyrus	-50	-42	0
Middle temporal gyrus	46	-58	4
Parahippocampal gyrus	-26	-40	-8
Parahippocampal gyrus	-12	-40	0
Parahippocampal gyrus	18	-46	-10
Inferior parietal lobe	-54	-22	44
Inferior parietal lobe	-32	-46	48
Angular gyrus	-46	-60	20
Angular gyrus	44	-72	28
Precuneus	-16	-76	34
Fusiform gyrus	26	-60	-10
Lingual gyrus	-16	-52	-2
Lingual gyrus	20	-66	2
Lingual gyrus	-16	-72	-8
Lingual gyrus	26	-80	-16
Lingual gyrus	20	-86	-2
Lingual gyrus	8	-92	-8
Cuneus	-18	-68	4
Cuneus	6	-72	24
Cuneus	8	-72	10
Cuneus	16	-76	30
Cuneus	28	-76	26
Cuneus	-8	-80	8
Cuneus	-2	-82	22

Cuneus	6	-82	6
Cuneus	16	-88	36
Cuneus	24	-88	24
Cuneus	-14	-90	32
Cuneus	-24	-92	18
Middle occipital gyrus	42	-66	-8
Middle occipital gyrus	40	-72	14
Middle occipital gyrus	-42	-74	0
Middle occipital gyrus	-46	-76	-10
Middle occipital gyrus	-32	-80	-14
Middle occipital gyrus	36	-82	2
Middle occipital gyrus	36	-84	12
Middle occipital gyrus	-26	-90	4
Inferior occipital gyrus	42	-78	-12
Inferior occipital gyrus	-40	-88	-6
Inferior occipital gyrus	26	-98	-14
Anterior cingulate cortex	-8	48	24
Midcingulate cortex	-14	-18	40
Medial frontal gyrus	-6	-20	66
Medial frontal gyrus	2	-28	60
Inferior frontal gyrus	34	54	-18
Inferior frontal gyrus	-46	10	24
Posterior insula	36	-10	14
Posterior insula	44	-22	20
Precentral gyrus	52	-6	32
Precentral gyrus	66	-8	24

Precentral gyrus	-56	-10	12
Precentral gyrus	-52	-10	24
Precentral gyrus	-50	-12	34
Postcentral gyrus	50	-20	42
Postcentral gyrus	48	-30	48
Superior temporal gyrus	58	-16	8
Superior temporal gyrus	-38	-34	16
Superior temporal gyrus	-56	-40	14
Middle temporal gyrus	52	6	-30
Middle temporal gyrus	52	-2	-16
Middle temporal gyrus	-56	-12	-10
Middle temporal gyrus	-50	-42	0
Middle temporal gyrus	46	-58	4
Parahippocampal gyrus	-26	-40	-8
Parahippocampal gyrus	-12	-40	0
Parahippocampal gyrus	18	-46	-10
Inferior parietal lobe	-54	-22	44
Inferior parietal lobe	-32	-46	48
Angular gyrus	-46	-60	20
Angular gyrus	44	-72	28
Precuneus	-16	-76	34
Fusiform gyrus	26	-60	-10
Lingual gyrus	-16	-52	-2
Lingual gyrus	20	-66	2
Lingual gyrus	-16	-72	-8
Lingual gyrus	26	-80	-16

Lingual gyrus	20	-86	-2
Lingual gyrus	8	-92	-8
Cuneus	-18	-68	4
Cuneus	6	-72	24
Cuneus	8	-72	10
Cuneus	16	-76	30
Cuneus	28	-76	26
Cuneus	-8	-80	8
Cuneus	-2	-82	22
Cuneus	6	-82	6
Cuneus	16	-88	36
Cuneus	24	-88	24
Cuneus	-14	-90	32
Cuneus	-24	-92	18
Middle occipital gyrus	42	-66	-8
Middle occipital gyrus	40	-72	14
Middle occipital gyrus	-42	-74	0
Middle occipital gyrus	-46	-76	-10
Middle occipital gyrus	-32	-80	-14
Middle occipital gyrus	36	-82	2
Middle occipital gyrus	36	-84	12
Middle occipital gyrus	-26	-90	4
Inferior occipital gyrus	42	-78	-12
Inferior occipital gyrus	-40	-88	-6
Inferior occipital gyrus	26	-98	-14

Healthy controls vs. Relatives of suicide victims, 2nd component, p = 0.02, FWER			
**Node**	**MNI coordinate**		
	**x**	**y**	**z**
Anterior cingulate cortex	-10	26	24
Anterior cingulate cortex	-2	38	36
Anterior cingulate cortex	10	22	28
Medial frontal gyrus	6	64	22
Medial frontal gyrus	6	8	50
Middle frontal gyrus	42	0	48
Middle frontal gyrus	20	-8	64
Superior frontal gyrus	-16	-4	70
Superior frontal gyrus	14	-2	70
Superior temporal gyrus	50	8	-2
Putamen	14	4	8
Putamen	-16	4	8
Putamen	28	0	4
Putamen	-22	8	-4
Putamen	30	-14	2
Putamen	24	10	2
Thalamus	6	-24	0
Thalamus	-2	-14	12
Thalamus	12	-18	8
Thalamus	-6	-28	-4
Hippocampus	28	-18	-16

Table S4: MNI coordinates of significant group differences between relatives of depressed patients with no family history of suicidal behavior and relatives of suicide victims are presented for the Network-Based Statistics (NBS) analyses at the whole-brain level.

**Relatives of depressed patients vs. Relatives of suicide victims, p = 0.02, FWER**			
**Node**	**MNI coordinate**		
	**x**	**y**	**z**
Inferior frontal gyrus	36	32	-2
Medial frontal gyrus	10	-18	74
Medial frontal gyrus	-12	-18	74
Medial frontal gyrus	2	-28	60
Medial frontal gyrus	6	8	50
Medial frontal gyrus	4	-18	58
Anterior cingulate cortex	-2	38	36
Midcingulate cortex	-14	-18	40
Midcingulate cortex	0	-14	46
Superior temporal gyrus	-38	-34	16
Superior temporal gyrus	52	-32	8
Middle temporal gyrus	46	-58	4
Posterior insula	36	-10	14
Posterior insula	36	0	-4
Posterior insula	44	-22	20
Precentral gyrus	-6	-20	66
Precentral gyrus	20	-28	60
Postcentral gyrus	-22	-30	72
Postcentral gyrus	28	-40	60

Postcentral gyrus	-20	-32	60
Superior parietal lobe	-30	-44	60
Thalamus	-2	-14	12
Thalamus	12	-18	8
Fusiform gyrus	-46	-50	-20
Middle occipital gyrus	-52	-64	6
Cerebellum	-16	-66	-20

Table S5: MNI coordinates of the 262 independent anatomical regions of interest used in the present study are presented, which were defined based on the extensively validated parcellation system provided by Power et al. [38] .

X	Y	Z
-24	-98	-12
26	-98	-14
24	32	-18
32	38	-12
-8	-52	60
-14	-18	40
0	-14	46
10	-2	44
-6	-20	66
-6	-34	72
14	-32	74
-54	-22	44
28	-16	70
10	-46	72
-22	-30	72
-40	-20	54
28	-40	60
50	-20	42
-38	-28	70
20	-28	60
44	-8	56
-30	-44	60
10	-18	74
22	-42	68
-46	-32	46
-20	-32	60
-12	-18	74
42	-20	54
-38	-14	68
-16	-46	74
2	-28	60
4	-18	58
38	-18	44
-50	-12	34
36	-10	14
52	-6	32
-52	-10	24
66	-8	24
-2	2	54
54	-28	34
20	-8	64
-16	-4	70
-10	-2	42
36	0	-4
14	-2	70

6	8	50
-44	0	8
50	8	-2
-34	4	4
-52	8	-2
-6	18	34
36	10	2
32	-26	12
66	-34	20
56	-16	8
-38	-34	16
-60	-26	14
-50	-26	6
44	-22	20
-50	-34	26
-52	-22	22
-56	-10	12
56	-6	14
60	-18	28
-30	-28	12
-40	-76	26
6	66	-4
8	48	-16
-12	-40	0
-18	64	-10
-46	-60	20
44	-72	28
-44	12	-34
46	16	-30
-60	-22	-18
-58	-26	-14
28	16	-16
-44	-64	34
-40	-74	44
-6	-54	28
6	-58	36
-12	-56	16
-2	-48	12
8	-48	30
16	-64	26
-2	-36	44
10	-54	18
52	-60	36
24	34	48
-10	40	52
-16	28	54
-36	20	50
22	40	38
12	54	38
-10	54	38
-20	46	40
6	54	16
6	64	22

-8	50	-2
8	54	4
-4	44	-10
8	42	-6
-12	44	8
-2	38	36
-2	42	16
-20	64	20
-8	48	24
64	-12	-20
-56	-12	-10
-58	-30	-4
64	-30	-8
-68	-42	-6
14	30	58
12	36	20
52	-2	-16
-26	-40	-8
26	-40	-12
-34	-38	-16
28	-76	-32
52	6	-30
-52	2	-28
46	-50	28
-50	-42	0
-30	18	-18
-2	-34	32
-6	-72	42
12	-66	42
4	-48	50
-46	32	-14
-10	10	66
50	36	-12
8	-92	-8
18	-92	-14
-12	-94	-12
18	-46	-10
40	-72	14
8	-72	10
-8	-80	8
-28	-80	20
20	-66	2
-24	-92	18
26	-60	-10
-16	-72	-8
-18	-68	4
42	-78	-12
-46	-76	-10
-14	-90	32
16	-88	36
28	-76	26
20	-86	-2
16	-76	30

-16	-52	-2
42	-66	-8
24	-88	24
6	-72	24
-42	-74	0
26	-80	-16
-16	-76	34
-2	-82	22
-40	-88	-6
36	-84	12
6	-82	6
-26	-90	4
-32	-80	-14
36	-82	2
-44	2	46
48	24	26
-46	10	24
-52	-48	42
-22	10	64
58	-52	-14
24	44	-16
34	54	-18
-22	40	-20
-18	-76	-24
16	-80	-34
34	-68	-34
48	10	32
-42	6	32
-42	38	22
38	44	16
50	-42	46
-28	-58	48
44	-52	46
32	14	56
38	-64	40
-42	-54	44
40	18	40
-34	54	4
-42	46	-2
34	-54	44
44	50	-2
-42	24	30
-2	26	44
10	-38	50
56	-44	36
42	0	48
32	32	26
48	22	10
-36	20	0
36	22	2
36	32	-2
34	16	-8
-10	26	24

0	14	44
-28	52	22
0	30	28
6	24	38
10	22	28
32	56	14
26	50	26
-40	50	18
2	-24	30
6	-24	0
-2	-14	12
-10	-18	8
12	-18	8
-6	-28	-4
-22	8	-4
-16	4	8
30	-14	2
24	10	2
28	0	4
-32	-12	0
14	4	8
8	-4	6
54	-42	22
-56	-50	10
-56	-40	14
52	-32	8
52	-28	-4
56	-46	12
52	32	0
-50	26	0
-16	-66	-20
-32	-56	-26
22	-58	-24
0	-62	-18
32	-12	-34
-32	-10	-36
10	-62	60
-52	-64	6
-46	-50	-20
46	-46	-16
48	-30	48
22	-64	48
46	-58	4
26	-58	60
-32	-46	48
-26	-70	36
-32	-2	54
-42	-60	-8
-16	-58	64
28	-4	54
-22	-16	-16
28	-18	-16
-20	-6	-20

22	-2	-20
-24	-34	-16
24	-30	-14
0	-30	-22
0	-24	-14
